# Power determination in vitamin D randomised control trials and characterising factors affecting it through a novel simulation-based tool

**DOI:** 10.1038/s41598-021-90019-7

**Published:** 2021-05-24

**Authors:** Jason Wyse, Rebecca Mangan, Lina Zgaga

**Affiliations:** 1 Discipline of Statistics and Information Systems, School of Computer Science and Statistics, Trinity College Dublin, College Green, Dublin 2, Ireland; 2 Discipline of Public Health and Primary Care, Institute of Population Health, Trinity College Dublin, Tallaght Cross, Tallaght, Dublin 24 Ireland

**Keywords:** Biomarkers, Diseases, Endocrinology, Medical research, Risk factors

## Abstract

Thousands of observational studies have linked vitamin D deficiency with numerous diseases, but randomised controlled trials (RCTs) often fail to show benefit of supplementation. Population characteristics and trial design have long been suspected to undermine power but were not systematically investigated. We propose a flexible generative model to characterise benefit of vitamin D supplementation at the individual level, and use this to quantify power in RCTs. The model can account for seasonality and population heterogeneity. In a simulated 1-year trial with 1000 participants per arm and assuming a 25-hydroxyvitamin D (25OHD) increase of 20 nmol/L due to the intervention, with baseline 25OHD in the population of 15, 35, 50, 60 and 75 nmol/L, the power to detect intervention effect was 77%, 99%, 95%, 68% and 19%, respectively. The number of participants required per arm to achieve 80% power according to baseline 25OHD of 15–60 nmol/L was 1200, 400, 600 and 1400, respectively. As expected, larger increases in 25OHD due to supplementation improved power in certain scenarios. For a population baseline of 50 nmol/L, with 1500 participants in each arm, there was 100% power to detect a 20 nmol/L 25OHD increase while it was 76% for a 10 nmol/L increase. Population characteristics and trial design, including temporal considerations, have a dramatic impact on power and required sample size in vitamin D RCTs.

## Introduction

A large number of observational studies have linked vitamin D deficiency with cancer, cognition, cardio-vascular, metabolic, autoimmune, infectious diseases, mortality, and many other illnesses^1^; most recently vitamin D has been implicated in COVID-19 infection and severity^2,3^. Vitamin D deficiency is very common world-wide: it is estimated that over 1 billion people are vitamin D deficient^4^. If disease associations are real, tackling deficiency could have an enormous impact on public health globally. Therefore it is not surprising that there has been a considerable interest in vitamin D in the last two decades. However, randomised controlled trials (RCTs) often fail to show benefit of vitamin D supplementation.

Vitamin D status is assessed by measuring 25-hydroxyvitamin D (25OHD) concentration in the circulation: levels below 25 nmol/L are generally considered to indicate “deficiency”, levels between 25 and 50 nmol/L “inadequacy”, and above 50 nmol/L “sufficiency”^5^. However, these cut-offs are still under debate and some advocate much higher levels for optimal health^6^. Interestingly, there might not be a single definition of vitamin D deficiency. To prevent rickets, levels of 50 nmol/L are “sufficient”^7^, but much higher concentrations may be required for other conditions. For example, levels of 100–150 nmol/L may be needed for prevention of cancer^8^ or multiple sclerosis^9^.

It is well-documented, and unsurprising, that the benefit of vitamin D supplementation is greatest in deficient individuals. In the context of RCTs where no benefit was found, common concerns invariably relate to the study population being vitamin D sufficient at baseline (limiting the extent of benefit supplementation could achieve), or treatment being too short or dose too low to have a meaningful impact on vitamin D status.

In addition to this, we note that an individual’s vitamin D status is determined primarily by exposure to ultraviolet B (UVB) radiation exposure in their environment^10,11^, and hence follows a strong seasonal pattern^12^. Given that an individual will benefit most from supplementation when they are deficient, any potential usefulness of vitamin D in prevention will also be affected by this seasonality: while supplementation may contribute the majority of vitamin D in winter, the same treatment may be dwarfed by the abundance of skin-synthesised vitamin D in the summer, as it has been reported that 30 min of whole skin surface exposure to summer sunshine is equivalent to 10,000–20,000 IU, even in Norway^13^. The impact of season on vitamin D status^14,15^ and association with disease traits^16–18^ is well established and lifestyle factors may strongly affect this^19^ or even reverse it^20^. Thus, the supplementation dose, duration and time of year in which a trial is carried out, as well as individual baseline status and other factors, may impact the comparisons of supplemented and placebo arms in trials^21^.

When planning a trial, one hopes at the very least that the assumptions made for the power calculation will roughly approximate the conditions expected in the wild, so that conclusions from analysed trial data can be made with a high degree of confidence. With regard to vitamin D, our claim is that obtaining the power for comparisons of supplementation schemes is non-trivial, and hence traditional RCT planning protocols cannot represent the specific nature of vitamin D trials. As is widely applied in biomedical research, power calculations are based on detection of an expected difference between groups (e.g. means) and variation in outcomes. Elicitation of this variation in a vitamin D context is challenging because variation within a subject (e.g. across seasons) is compounded with variation between subjects (e.g. across trial arms), the impact of which has also been observed in vitamin D RCTs^11^. We advocate exploring variation in a different way. Frameworks such as the DELTA$^2$ guidance on choosing the target difference for RCTs^22^ highlight the importance of appropriate elicitation of effect sizes for sample size and power calculations. We demonstrate through a model characterisation of vitamin D supplementation, that effect sizes are not easily articulated in the context of vitamin D trials. The simulation tools our proposal offers, allow a gateway to reclaiming prior knowledge and intuition for designing complex trials where there is a strong temporal within and between subject variability. Obtaining power through simulation is a common approach for complex study designs, see for example^23,24^. This is critical, as design decisions can have detrimental impact on the reliability of RCT conclusions in the context of vitamin D^25^. Given the widespread interest in vitamin D, a bespoke flexible planning tool is timely, both for ensuring adequate sample size in future trials, and better informed interpretation of reported findings. This is particularly relevant for trials that found no benefit, as the likelihood of a false negative results can be interrogated.

In this paper, we develop a simulation-based approach for sample size and power calculation in RCTs of vitamin D supplementation, and we show the impact of population characteristics and trial design on the power. By simulating individual vitamin D status trajectories, dominant sources of variability and heterogeneity in status (and consequently potential benefit of supplementation) can be accounted for. Each component of the simulation model can be parameterised to harness any available domain expertise (e.g. cancer or bone researchers may define “optimal” vitamin D status differently), specific characteristics of the population (e.g. baseline level), and trial-related factors (e.g. duration or the increase in 25OHD in treatment group). The tools discussed in this paper are easily used through the R package SimVitD^26^ available on the Comprehensive R Archive Network (CRAN).

For the sake of exposition, the paper considers a respiratory infection. Simulation of exposures at an individual level are used to approximate the potential effect of supplementation in protecting against these, accounting for natural cyclic patterns in status. The power is approximated via simulation and this can be used to determine the required sample size to obtain a given power. The text focuses on vitamin D, however the methods presented have the potential to be quite flexible and could be extended to other nutrient studies where the nutrient level varies according to some pattern e.g. vitamin $\mathrm {B}_{12}$ shots to supplement vegetarian diets, or in prevention of thromboembolic or bleeding events in patients who are on vitamin K antagonists and other.

## Methods

### Modelling individual vitamin D status trajectories

As most of an individual’s vitamin D is derived from synthesis in skin following UVB exposure^10,11^, vitamin D status will naturally vary throughout the year. 25OHD concentration (marker of vitamin D status) tends to peak late in the summer, following the period with the strongest UVB radiation; the status trough will follow the period of lowest exposure^27,28^. It is useful to note that the peak and trough in an individual’s status will depend on geolocation; there will be variability within, say, the northern hemisphere^28^. This paper works on the assumption of a northern hemisphere seasonal schedule with summer months being June to August. Cyclic status profiles follow the assumed yearly periodic curve with a trough in February or March and peak in August or September^27,28^. The peak 25OHD occurs with 1–2 month time lag following the peak UVB radiation (here we assume 2 months); this reflects the period of pronounced vitamin D accumulation arising from abundant production of the nutrient in the skin.

Consider a group of trial participants, indexed by *i*. A phase shifted cosine curve with a lower threshold is used to model a participant’s vitamin D status^12^ derived from non-supplement sources (UVB) over time,

1 $$\begin{aligned} V_i^{\mathrm{pl}}(t) = \max \left\{ \mu _i + H_i + A_i \cos (2\pi t - \nu ) \, , \, 10\right\} , \quad t \ge 0. \end{aligned}$$

Here, $\mu _i$ is a mean level that could be described by participant specific characteristics i.e. $\mu _i = \mathbf {x}_i^{\mathrm{T}} {\varvec{\beta }}$ with $\mathbf {x}_i$ a vector of covariates. Models of vitamin D involving parameterised cosine functions have been used previously to describe seasonal variation in 25OHD^12^, and this would appear a natural choice to characterise these seasonal patterns. UVB is by far the most dominant source of vitamin D, as food sources are largely scarce^29^. The lower threshold of 10 nmol/L of circulating 25OHD is a detectability threshold. In (1) *t* is time measured in years i.e. the interval [0, 1] corresponds to 1 year. The parameter $H_i$ gives a perturbation of the mean around $\mu _i$ similar to a random effect, and $A_i \ge 0$ controls the change in status between periods with and without significant UVB exposure. The phase adjustment $\nu$ accounts for a lag effect from UVB exposure to expressed circulating vitamin D level. This can be used to make adjustments for geolocation effects e.g. northern/southern hemisphere. Here, $\nu =\pi$ is assumed, meaning that time is counted from the beginning of March $(t=0)$, and at that point 25OHD is lowest.

**Figure 1:**
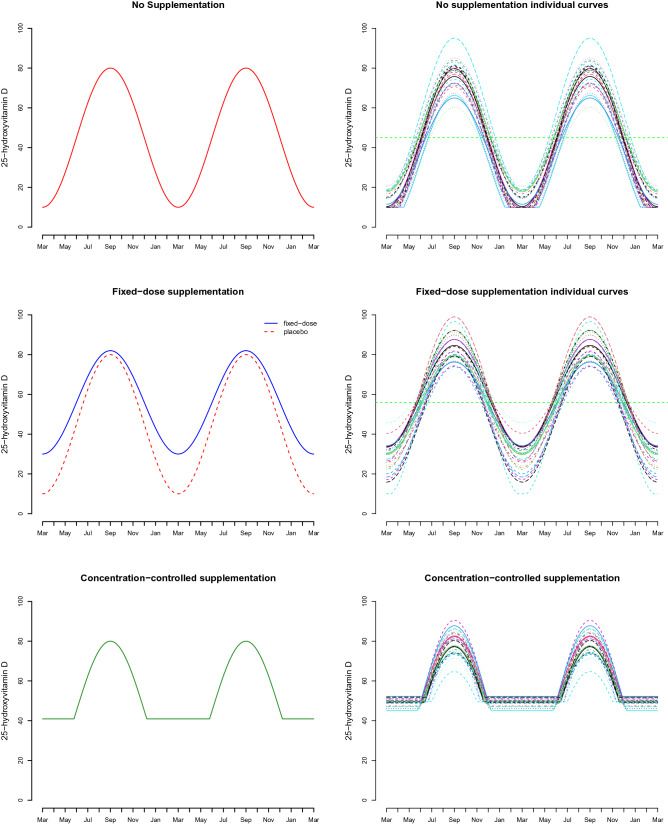
25-hydroxyvitamin D (25OHD) status profiles for placebo, fixed-dose supplementation and concentration-controlled intervention schemes. The middle panel on the left compares the fixed-dose scheme with more uptake during deficient periods. Horizontal green dashed lines in the top two panels indicate effective overall mean levels of 25OHD assumed in the model.

Variation in individual 25OHD concentration is accounted for by generating the amplitude and a height perturbation in (1) via

$$\begin{aligned} A_i \sim \text {Gamma}\left( \alpha _A, \beta _A \right) \quad H_i \sim \text {N}\left( 0, \sigma _H^2 \right) , \end{aligned}$$

independently drawn for each individual. The shape and rate parameters $\alpha _A, \beta _A$ are chosen to have a specified expected value and standard deviation $\mu _A, \sigma _A$. The height perturbation standard deviation $\sigma _H$ will also be specified. These specifications should be made to sufficiently represent typical population variation in trial participants. The top panel of Fig. 1 shows what may be representative of a target cohort.

### Intervention: randomised controlled trials (RCT) and randomised concentration-controlled trials (RCCT)

A number of possible approaches may be under consideration when planning a prospective trial. The curve in (1) corresponds to no supplementation and is referred to as *placebo* in what follows. Two potential supplementation schemes are considered in this paper and determined by the nature of the trial. The first of these, an RCT, would be the more common approach. The second, RCCT, gives an example of frequent 25OHD measurement during the trial and a responsive dosing scheme design that one might employ had they the necessary resources.

#### Fixed-dose scheme

A fixed-dose scheme corresponds to an individual taking a daily supplement of a fixed amount, and this is the prevailing approach in RCTs. Supplementation might provide more of a boost when vitamin D levels are low. To allow for this, the no supplement curve is modified by adding a flexible function $F_i(t)$

$$\begin{aligned} V_i^{\mathrm{fix}}(t) = V_i^{\mathrm{pl}}(t) + F_i(t). \end{aligned}$$

where

$$\begin{aligned} F_i(t) = \delta _i \,\left[ \omega _i + \frac{1}{2}(1-\omega _i) \,\left( 1 + \sin \left( 2\pi t- \frac{\pi }{2} - \nu \right) \right) \right] \quad \omega _i \in [0,1]. \end{aligned}$$

Here $\delta _i$ represents an individual’s overall derived benefit from that dosage, accounting for variation in overall assimilation. The parameter $\omega _i$ gives the proportion of the fixed-dose which is always utilised. As supplementation may have more impact in periods of deficiency, we allow the uptake from the remaining $1-\omega _i$ proportion of the fixed dose to vary according to a complementary cosine function. Individual $\omega _i$ values are sampled from a beta distribution with expected value $\mu _\omega$ and standard deviation $\sigma _\omega$. The value of $\delta _i$ may also be simulated at an individual level using a truncated distribution capped at the administered dose level. A distribution for this purpose is given in the supplementary material (S.2).

#### Concentration-controlled scheme

A concentration-controlled scheme allows an individual to be monitored regularly and their status kept above a 25OHD threshold $\rho _i$, for example in a randomised concentration-controlled trial (RCCT) design^30^. That is

$$\begin{aligned} V_i^{\mathrm{dyn}}(t) = \max \left\{ \, \rho _i, V_i^{\mathrm{pl}}(t)\,\right\} . \end{aligned}$$

A comparison of the placebo, fixed-dose supplementation and concentration-controlled schemes can be made from Fig. 1. The individual target level is simulated via $\rho _i \sim \text {Gamma}\left( \alpha _{\rho },\beta _{\rho }\right) ,$ with $\alpha _{\rho },\beta _{\rho }$ determined to give a specified expectation $\mu _{\rho }$ and standard deviation $\sigma _{\rho }$.

### A model for benefit of vitamin D supplementation

Individuals’ exposures (e.g. to infection, or other risk factors) are assumed to arise independently from their vitamin D status profiles. Benefit of supplementation is assumed here to correspond to heightened immune defences, giving protection against illness. The proposed generative model for supplementation benefit is:

1. simulation of an individual’s vitamin D status profile (introduced in “Modelling individual vitamin D status trajectories”)
2. simulation of times of exposure (to infection in this example)
3. get probability of developing illness conditional on vitamin D status at exposure
4. simulation of occurrence of event (illness) using the result of step 3.

#### Exposures to infection

**Figure 2:**
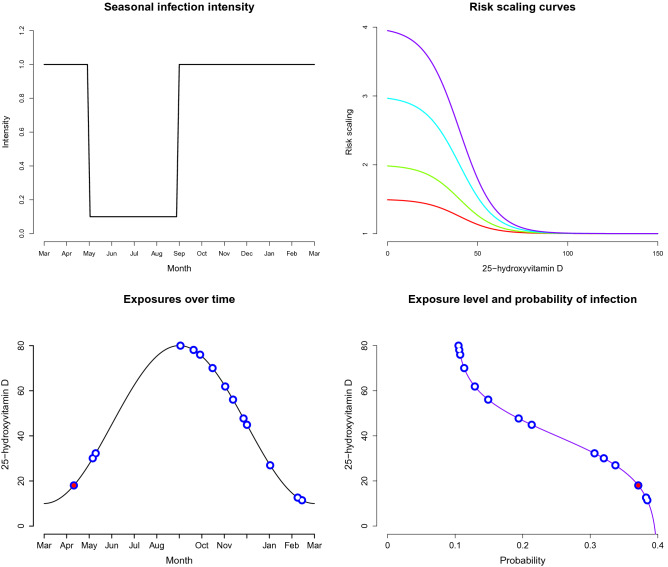
Top row left: intensity function for a seasonal infection shown in units of exposures per week. Top row right: generalised logistic curves giving the risk scalings for $l=1$ and $u=1.5,2,3,4$. Bottom row left: example of an individual’s exposures shown in blue, with red centre indicating an infection developed following exposure. Bottom row right: corresponding 25OHD status at exposure, shown as a function of the probability of infection for that status level.

Exposure to infection is taken as the lead example in this paper (one could, for example, be examining protection against allergic reaction following exposure to allergens, asthma attack, relapse of autoimmune disease). An individual’s exposures over the period of a prospective trial are simulated from a Poisson process. In the case of seasonally concentrated infections (e.g. flu, see Fig. 2), a non-homogeneous Poisson process (NHPP) with rate function $\lambda (t), t\ge 0$ is used^31^. Simulations from an NHPP can be carried out conveniently in R using the poisson^32^ package. The function $\lambda (t)$ can represent different kinds of exposure (for example, respiratory infections are common in winter; pollen allergies in late spring and summer). It can also, of course, represent a constant rate of exposure. Instituting this function is an opportunity to incorporate domain expertise into the planning stage of the trial. Modelling exposures in this way would appear reasonable considering the varying social mixing and other lifestyle habits and individual circumstances that participants might typically have. In general, the expected number of exposures over a time window $(0,\tau ]$ is given by $\int _{0}^{\tau } \lambda (t)\, \text {d} t$. For step intensity functions where $\lambda (t)=\lambda _0$ for some interval of *t* values, this can conveniently be re-scaled to an exposures-per-week rate over that interval.

#### Likelihood of infection

The likelihood an individual contracts infection after exposure depends on the vitamin D status at exposure. This is modulated by a baseline prevalence $p_0$ and a relative risk scaling curve. As one’s status moves towards insufficient from sufficient 25OHD concentration, their risk increases. Whether an individual contracts infection from a given exposure is assumed to be independent of other exposures conditional on the individual’s status curve. A sigmoid shaped dose–response curve is justified in studying effectiveness of nutrients^33^, including vitamin D. This thinking is adopted through the risk scaling curve which is taken as a member of the generalised logistic family

$$\begin{aligned} g(x) = l + \frac{u - l}{1 + \mathrm {e}^{a + b \,x}}, \end{aligned}$$

where *x* represents status. The parameters *l*, *u* give the lowest and highest relative risk scaling values. The difference $u-l$ represents how much more likely one is to get infection with completely insufficient 25OHD compared to being fully sufficient. The values of *a* and *b* are determined by providing reference values between which one observes the steepest change of the relative risk scaling curve. The points 10 and 70 nmol/L are used for the results presented in this paper; these are the status levels between which one sees the greatest change in protection attributable to vitamin D. See Fig. 2 for an example showing scaling curves where the probability would vary between $p_0$ and 1.5, 2, 3, 4 times $p_0$. The probability $p_0$ gives the probability of a fully sufficient vitamin D individual contracting infection after exposure. There is much debate around the reference values chosen here as 10 and 70 nmol/L and this is another opportunity to explicitly represent domain experience in trial planning.

#### Summary of simulation steps

Before progressing further, we give an overall summary of the generative model of exposures and infections. Consider individual *i* and let $T_{1},\dots ,T_{M}$ denote the times at which they are exposed. Exposure times only within the time frame of the trial are used: $\tau _{\mathrm{start}} < T_k \le \tau _{\mathrm{end}}$, $k=1,\dots , M$.

$$ \begin{array}{*{20}l}  {T_{1} , \ldots ,T_{M} \sim {\text{NHPP}}(\lambda (t))} \hfill & {{\text{simulate}}\;{\text{individual's}}\;{\text{exposure}}\;{\text{times}}} \hfill \\  {L_{k} = V_{i} (T_{k} )} \hfill & {{\text{find their status }}k = 1, \ldots , M} \hfill \\  {P_{k} = p_{0} g(L_{k} )} \hfill & {{\text{get the probability of infection after exposure }}k = 1, \ldots , M} \hfill \\  {I_{k} \sim {\text{Bernoulli}}(P_{k} )} \hfill & {{\text{simulate whether infection is developed from exposure }}k = 1, \ldots ,M.} \hfill \\  \end{array} $$

In the case of infections ($I_k=1$), one may also wish to impose a non-susceptible period, for example, an exponentially distributed amount of time where the infected individual is not susceptible to a new infection. The package SimVitD provides this option. S.3 in the supplementary material gives a glossary of all parameters involved in the simulation.

### Power of detecting benefit of supplementation

Determining whether there is a benefit of supplementation will ordinarily be carried out by investigation of the number of events (e.g. infections) that occurred in participants assigned to each arm over the trial duration, or some function thereof.

#### Types of comparisons

Define

$$\begin{aligned} \theta _s= & {} \Pr \{ \text {individual gets } \ge 1\text { infection in arm } s \}\\ \mu _s= & {} \text { expected number of infections for individual in arm } s \end{aligned}$$

The power of the tests

2 $$\begin{aligned} H_0: \theta _{\mathrm{pl}} \le \theta _{\mathrm{supp}}&\quad&H_A: \theta _{\mathrm{pl}} > \theta _{\mathrm{supp}} \end{aligned}$$

3 $$\begin{aligned} H_0: \mu _{\mathrm{pl}} \le \mu _{\mathrm{supp}}&\quad&H_A: \mu _{\mathrm{pl}} > \mu _{\mathrm{supp}} \end{aligned}$$

will be of interest.

##### Theorem 1

*Consider a two-armed vitamin D trial, where individuals in the first arm receive a placebo and those in the second all receive an intervention (supplement) following the same scheme (e.g. all fixed dose). Then making the same assumptions as outlined in* “Modelling individual vitamin D status trajectories” *and* “A model for benefit of vitamin D supplementation” *we have that:*

1. *the propensities to contract at least one infection in each group satisfy* $\theta _{\mathrm{pl}} >\theta _{\mathrm{supp}}$
2. *the expected number of infections in the placebo group is larger than that in the supplement group*, $\mu _{\mathrm{pl}} >\mu _{\mathrm{supp}}.$

*That is, the simulation model in* “Modelling individual vitamin D status trajectories” *and* “A model for benefit of vitamin D supplementation” *will correctly generate samples under* $H_A$ *for* (2) *and* (3) *when there is indeed an intervention (supplement) administered*.

A proof is provided in the supplementary material. This result says that if one considers the proposed models to be reasonable for the purposes of study planning, then approximating the power using them should serve as a proxy for a trial in the wild.

To approximate the power of detecting treatment effects, tests of (2) and (3) are carried out using a non-parametric Bootstrap^34^. Two sample tests from the **R** package **wBoot**^35^ are used. These comparisons are extensible to other planning scenarios. For example, to account for age differences, one could have an age range specific logistic curve when carrying out the simulation steps in “Summary of simulation steps”. Then a logistic regression incorporating age range could be used to approximate the power of any comparisons. Again, there is scope for domain knowledge to be incorporated in such considerations.

Traditional power calculations return a sample size for a specified effect size, Type I error and target power. Here the concept of a catch-all prescriptive effect size cannot be characterised directly through a univariate quantity. It will depend on dosage and risk scaling. However, an implied effect size for tests (2) and (3) can be approximated and returned as a by-product of our approach.

#### Approximating power

**Figure 3:**
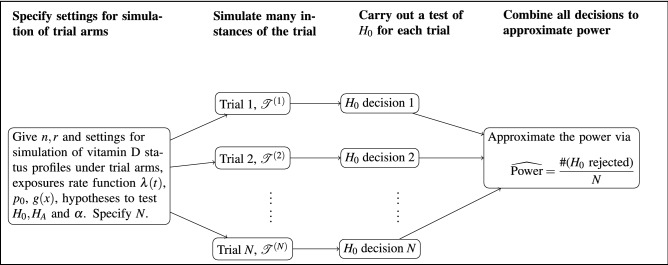
Simulation process for estimating the power of a study.

Let there be $n_{\mathrm{pl}} = n$ and $n_{\mathrm{supp}} = r n$ participants in each of the two arms “$\text {pl}$” and “$\text {supp}$”. Here the supplement arm will be of size *r* : 1 to the placebo arm. Often $r\ne 1$ will be investigated and in cases where necessary $n_{\mathrm{supp}} = \lfloor r n \rfloor$. Power to detect group differences is approximated by simulating the trial a large number of times using the models outlined in “Modelling individual vitamin D status trajectories” and “A model for benefit of vitamin D supplementation” and carrying out a bootstrap hypothesis test on each of these. Let ${\mathscr {T}}^{(1)}, \dots , {\mathscr {T}}^{(N)}$ denote *N* simulated trial realisations, each simulated under $H_A$ (i.e. where there is a difference). For each realisation, the test of hypothesis is applied to that data and the decision to reject or not is recorded. This gives the process illustrated in Fig. 3. Rejection in any case is based on a significance level $\alpha$.

A simulation-based estimate of the power is given by

$$\begin{aligned} \widehat{\text {Power}} = \frac{\sum _{j=1}^N \mathbb {I}(H_0 \text { rejected for } {\mathscr {T}}^{(j)})}{N}, \end{aligned}$$

the proportion of times $H_0$ was rejected when $H_A$ corresponds to the true generating process. Since trial instances are generated independently, the law of large numbers guarantees convergence in probability to the true power

$$\begin{aligned} \widehat{\text {Power}} \rightarrow \text {Power} \end{aligned}$$

as $N\rightarrow \infty$. The precision of the simulation based power estimate will scale as $O(N^{-1/2})$. The **R** package **SimVitD** implements this approximation for the comparisons outlined in “Types of comparisons” including convenient utilities for visualisation.

This procedure to estimate the power can be repeated for a range of possible values of *n* to construct a power function for the comparison. At first glance, this may appear to have a heavy computational load. Note however that these simulations may be easily parallelized using, for example, the **R** package **parallel**^36^. Also, the computations need only be carried out in the planning stages of a trial, and so any computational latency may not be a crucial issue.

### Examples

#### Examining the impact of baseline 25OHD concentration in the population and of average 25OHD change due to intervention

Here we examine the RCT power for a two-armed trial with five different populations having baseline 25OHD concentrations of: 15, 35, 50, 60, 75 nmol/L (in this context, this is annual average 25OHD in the population, i.e. the horizontal dashed green line in the top right panel of Fig. 1), and assuming minimum detectable levels of 10 nmol/L. This translates to $\mu =15, 35, 50, 60, 75$. The researchers aim to enrol a heterogeneous cohort of participants, so a large scatter around the expected maximum and minimum levels would be anticipated and is reflected as $\mu _A=15$ $\sigma _H=5$, $\sigma _A=5$. The primary endpoint is the number of infections contracted over the trial duration.

Participants receive either a placebo or a fixed-dose vitamin D supplement. For each population we run three trials: those in the intervention arm receive a dose that is equivalent to 10, 20 or 40 nmol/L increase in 25OHD, with little variability in the derived uptake (i.e. a large value of $\gamma$ is assumed and we take $\mu _\omega = 0.8, \sigma _\omega =0.1$, see supplementary material S.2.).

A seasonal infection is considered where the intensity function is expressed through a step function. The intensity is shown in Fig. 2 with its equivalent per week exposure rate during the corresponding period. That is having 1 expected exposure per week from September to April end, and 0.1 per week from May to August end. Two different levels of risk scaling are considered following “Likelihood of infection”. In each case $l=1$ and then we consider relative risk comparing highly insufficient and fully sufficient of either 2 or 4, i.e. $u=2,4$ (Fig. 2). The infection probability which gives the probability for a fully 25OHD sufficient individual becoming infected on exposure is taken to be $p_0=0.03$ in all cases. A non-susceptible period is simulated after each infection. This is exponentially distributed, with an expected duration of two weeks. Here the quantity to compare the two arms is the expected number of infections i.e. test (3) with $\alpha =0.05$. The power approximation uses $N=500$ simulations of each trial condition with 500 bootstrap replications for each hypothesis test. Each configuration is run five times independently to explore the Monte Carlo error.

**Figure 4:**
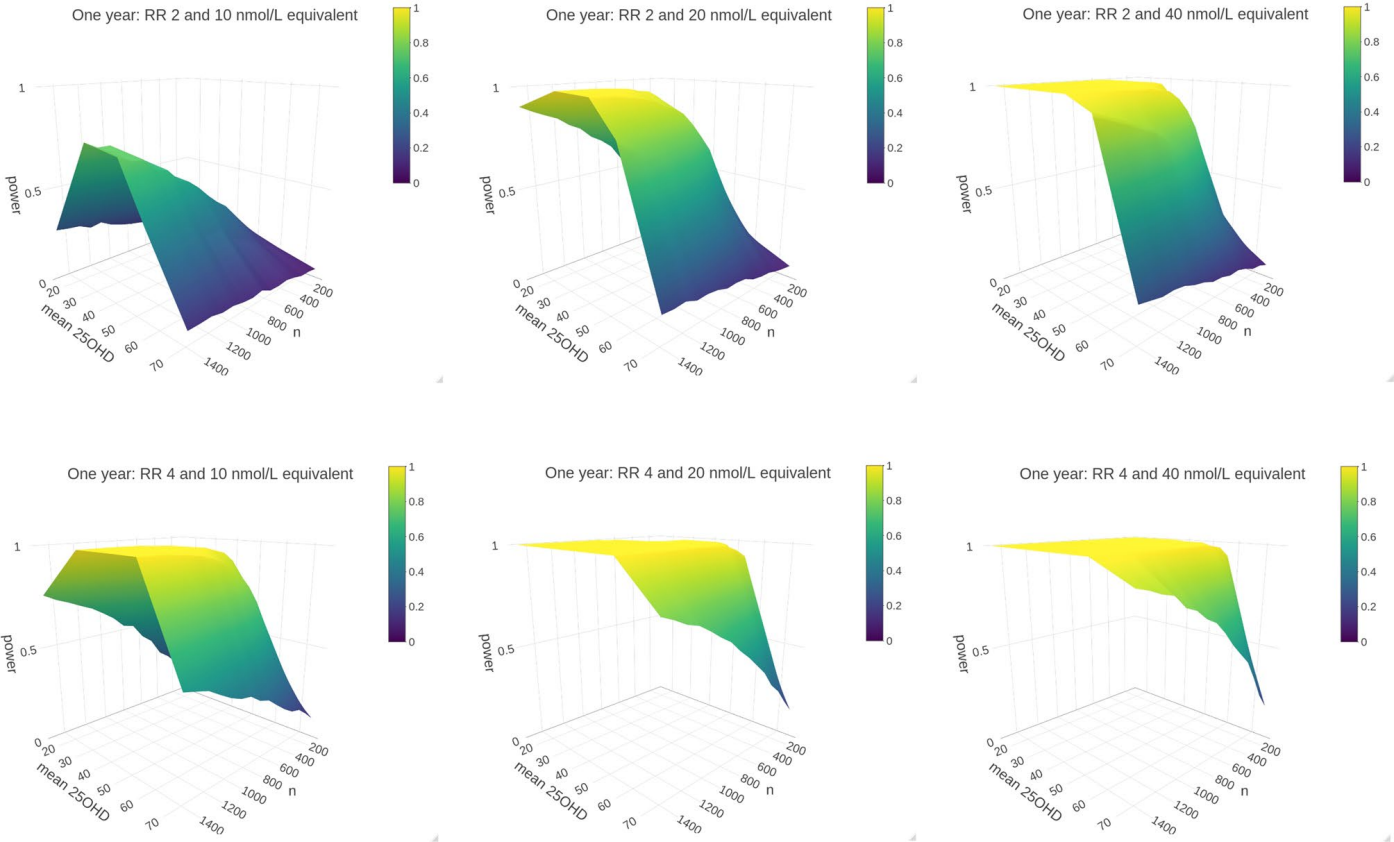
Power surfaces are shown for intervention that achieves a 25OHD increase of 10 nmol/L (left), 20 nmol/L (centre),and 40 nmol/L (right) versus placebo, against the sample size *n* and population baseline vitamin D status $\mu$. The top row shows surfaces when relative risk of 2 is assumed between fully insufficient and fully sufficient i.e. $u=2$, and the bottom row shows the same for $u=4$. Here *n* gives the number of participants in each trial arm.

Figure 4 shows power surfaces constructed by taking the Monte Carlo average over the five replications of each trial condition with $n=100,200,\dots ,1500$ in each trial arm ($r=1$).

#### Examining the impact of the start date of trials for 6 month trials

**Figure 5:**
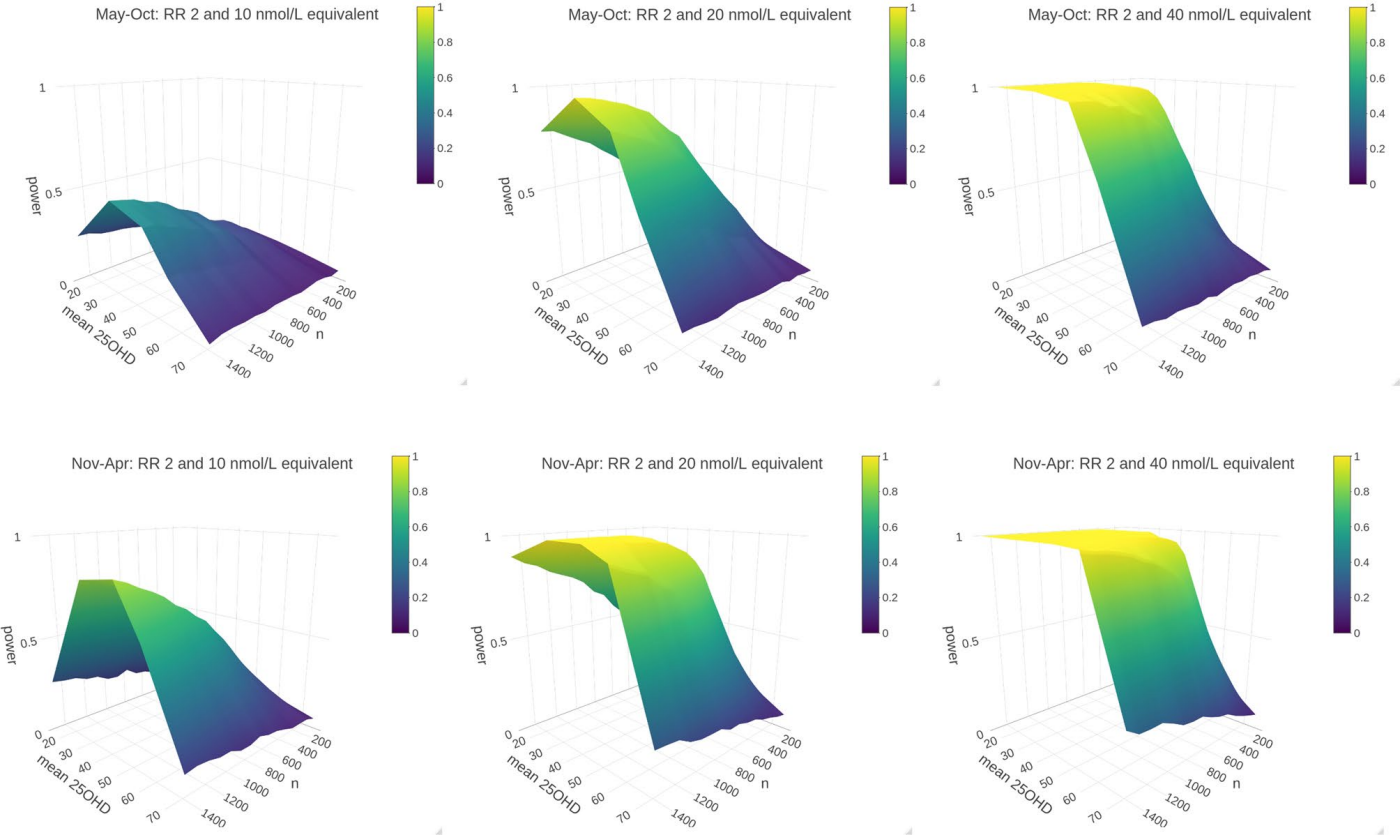
Six month trials. Top row: power surfaces for “summer trial”, running from May until end of October. Bottom row: “winter trial” running from November through April. All experiments assume relative risk between fully depleted and fully replete of 2, $u=2$. *n* represents the number of participants in each trial arm.

Since status level varies naturally throughout the calendar year, the date of trial initiation could conceivably have an impact on the trial power. Anecdotally, this has been observed, and a number of vitamin D trials have been conducted over winter months. To investigate this approach through our simulation model, we repeat the experiments above , but this time make the duration of the trial 6 months rather than 1 year and restrict our investigations to $u=2$. Two 6 month periods are considered; beginning of May to the end of October and beginning of November to the end of April. The estimated power surfaces are shown in Fig. 5.

## Results

As expected, we found that the trial power was very strongly linked with the relative risk scaling: at least 80% power was achieved in majority of experiments when relative risk of 4 ($u=4$) between fully sufficient and fully insufficient was assumed, but this was not the case when the relative risk was taken to be $u=2$. Details are given in the supplementary material (Section S.4, Tables S1–S5). The increase in 25OHD attributable to intervention also played a major role: sufficient power was not reached with an equivalent increase of 10 nmol/L in any of the test cases when $u=2$. For 20 and 40 nmol/L increases (Table 1; S.4 supplementary material), we observed a dramatic impact of population baseline 25OHD concentration. When annual average baseline 25OHD surpasses $\sim$ 50 nmol/L, the power deteriorates sharply. For example, while a trial with 1400 participants per arm (Table 1) would have close to full power to detect an intervention effect when the population baseline concentration is 35 nmol/L, the power would drop to 21.24% if this baseline was 75 nmol/L. Interestingly, for the $u=2$ case, we also have power plunges at 10 nmol/L intervention. This implies that the effect of intervention is less detectable when participants are at extreme ends of the 25OHD sufficiency spectrum.

**Table 1:** Power (in percentage) for 20 nmol/L equivalent dose at $u=2$ for a range of sample sizes and baseline population 25OHD concentrations.

*n* (per arm)	15 nmol/L	35 nmol/L	50 nmol/L	60 nmol/L	75 nmol/L
100	19.1	36.6	28.9	16.8	8.1
200	25.6	54.2	42.1	23.3	8.9
300	35.2	70.5	54.6	28.9	10.0
400	44.2	**81.8**	67.3	37.8	11.6
500	49.8	**88.2**	74.8	41.3	12.1
600	56.1	**93.3**	**81.8**	49.1	13.3
700	64.0	**96.6**	**87.6**	54.8	16.0
800	68.2	**97.3**	**90.5**	60.4	16.7
900	71.4	**98.7**	**92.8**	63.8	16.5
1000	76.5	**99.1**	**95.3**	67.5	18.5
1100	79.2	**99.5**	**96.8**	72.8	19.4
1200	**83.3**	**99.8**	**97.7**	75.1	19.2
1300	**85.5**	**99.9**	**98.6**	77.5	21.7
1400	**88.0**	**99.9**	**98.8**	**81.0**	21.2
1500	**90.8**	**100.0**	**99.1**	**84.6**	21.8

Designs having $\ge 80\%$ power are in bold font.

Figure 5 shows the estimated power surfaces for the 6 month trials. We note here that the power surface for the trial conducted from November to April is higher overall, even at the 10 nmol/L equivalent dose. The intervention effect is less detectable for those with high baseline vitamin D levels as noted previously.

## Discussion

This paper proposes a bespoke approach to establishing power and sample size required for randomised controlled trials investigating the benefit of vitamin D supplementation. The tools we present aim to account for dominant sources of variability and heterogeneity in vitamin D status that impact on the potential benefit of supplementation, and enable quantification of the impact different factors may have on power, an investigation that cannot be conducted in vivo. The specific issue with vitamin D is that 25OHD naturally fluctuates in both arms^11^. This makes articulation of a vitamin D supplementation effect more difficult since treatment effect can vary within and between individuals and is impacted by other factors (e.g. skin exposure to UVB).

Our results highlight four considerations regarding vitamin D RCTs. Firstly, how well powered a trial is depends strongly on the population that participants are drawn from; vitamin D deficient populations will have more detectable effects in certain instances. Secondly, even quite large increases in 25OHD in the intervention arm might not achieve sufficient power if the population is already 25OHD sufficient. Thirdly, trial power is closely connected with the expected magnitude of the effect of vitamin D, as illustrated by the different surface shapes in the top and bottom rows of Fig. 4. Finally, our results support benefit of conducting trials in the winter time as the start date of the trial has a demonstrable impact on the overall power. In practice, participants may be enrolled to such trials on an ongoing basis; what our simulations highlight is that the schedule for the roll-out of a trial lasting less than a year should be mindful of seasonal change.

The issue of high baseline 25OHD concentrations is an increasingly important consideration. Vitamin D supplementation is becoming more prevalent in general and in diseased populations^37,38^. Additionally, research ethics committees often request that potential trial participants who are vitamin D deficient should not be permitted to participate in the trial. Summarily, those who would benefit the most are excluded, and baseline 25OHD in the trial population is further increased artificially.

Two key aspects of the work need to be highlighted. First, as seasonal variation in solar radiation is pronounced, there will be a natural, cyclic variation in vitamin D status^12^; consequently, the relative contribution of supplementation to the overall vitamin D status, and it’s impact, will vary seasonally. Therefore, there may be a large within-person heterogeneity in the general effectiveness of supplementation depending on the time of year. Simulating individual trajectories allows for varied benefit of supplementation both between and within individuals; both are accounted for in the power approximations. Second, the relationship between nutrient status and health is often best represented by a sigmoid curve, and evidence suggests this might also be the case for vitamin D^33^. This implies that below a certain threshold further deterioration in status won’t further worsen health (fully insufficient), and similarly, above a certain threshold no extra benefit will be achieved (fully sufficient). The approach we propose allows investigators to hypothesise what these thresholds are. By modelling individual vitamin D trajectories, an individual’s relative risk can vary, as their 25OHD fluctuates between fully insufficient and fully sufficient. It is important to appreciate that within-arm the risk profile varies over the trial, and risk is not constant. In simple terms, this means that observations from a more deficient participant will contribute more to the study power, than observations from vitamin D sufficient participant.

After decades of research, the interest in vitamin D is not winding down, and the issue of vitamin D supplementation remains strongly divisive. Findings from trials are consequential. Our proposal provides a utility to aid sample size calculation in prospective vitamin D trials (and can be adapted for nutrients where status might change over time). The solution we give is a flexible model which characterises the main components of variability in a vitamin D trial, and key parameters can be chosen by the user and easily visualised. The findings we present are also relevant for post–hoc assessment of power for past trials (we provide an illustrative analysis in the supplementary material S.5)^39–42^. On foot of null-findings, treatment cannot be recommended and investment in future trials becomes less likely. Therefore, it is critical to appreciate the likelihood of false-negative findings. The key role of 25OHD concentration when evaluating trial findings has long been speculated. This was recently supported by findings from a large trial that found benefit in those who maintained high intra-trial 25OHD concentration^43^. Two other large trials found benefit of supplementation in normal weight individuals ( same dose of treatment achieves greater increase in 25OHD concentration in non-overweight or obese)^44,45^.

A number of studies have examined the protective properties of vitamin D in acute respiratory tract infections in different populations, as detailed in a thorough individual-patient data (IPD) meta-analysis^46^. The IPD meta-analysis found that vitamin D supplementation was effective at reducing the risk of these infections. However, the majority (16 out of 24) of original research studies included in this meta-analysis did not detect significant benefit of vitamin D (7 found significant protective effects and one concluded increased risk). In the light of these findings, it should be considered whether the null-findings from these 16 studies are false-negatives. Since it is reasonable to assume that investigators conducted sample size calculation prior to conducting a trial, it would be instructive to investigate the calculations deployed and their implied power.

A key novelty of our approach lies in modelling vitamin D status and exposures, and propagating these through the power calculation. Primary sources of variability in vitamin D trials are characterised in such a way as to allow domain expertise and knowledge of characteristics of the population trial participants to flexibly inform their parametrisation. In addition to looking purely at a traditional fixed-dose intervention (allowing the 25OHD response to treatment to vary between individuals), there is scope to investigate the concentration-controlled design aimed at achieving target 25OHD concentration in intervention arm. The power approximation presented relies on assuming that the model proposed for status and infections provides a reasonable approximation of a trial in the wild. If this is the case, the theorem of “Approximating power” justifies the approximation. The flexibility of the model allows for incorporation of potential covariate effects (e.g. BMI, skin tone) at the planning stage. If there are 25OHD characteristics that are known to impact specific groups of individuals in the cohort being targeted, then these effects can be included in the simulation model. As much information as is available at the time of trial conception can be incorporated into the power and sample size determination and this is particularly important in the case of vitamin D where extensive domain experience may be harnessed.

It is certain that individual 25OHD trajectories do not follow a smooth annual curve, as a number of factors might cause departures from this in either direction. For example, a sun holiday may boost vitamin D status or a surgery might deplete it^47^. However, the purpose of this model is to characterise the predominant sources leading to large variability in 25OHD level and treatment effect in vitamin D RCTs. What we propose is native to the cyclic variation expected in vitamin D. There are adjustments that would make the generative model of disease more realistic; for example, one may expect disease events within an individual to be correlated beyond just the vitamin D status curve. However, for the sake of planning, the model we propose should serve as a representative scheme of the phenomena one could conceive unfolding throughout a vitamin D trial. This study primarily considers outcomes that are acutely affected by contemporaneous vitamin D status. Many outcomes, for example cancer, take years to develop, and the role of vitamin D supplementation in such cases will be more difficult to model.

There is much interest in vitamin D supplementation as an inexpensive way to improve quality of life in general. Establishing evidence-based guidelines on the use of vitamin D supplements requires conclusions from appropriately powered trials. Recently impact of deficiency on the immune system is being debated with respect to COVID-19 infection. This work is timely in proposing power determination tools in this direction, where considerations about the natural cycle of solar radiation native to vitamin D uptake have been incorporated.

While developed for vitamin D trials, the strategy presented may be extended to other nutrient studies. For example, one could instead think of a status curve of the form

$$\begin{aligned} S_i(t) = \max \left\{ \mu _i + H_i + K_i\,t^q + A_i \cos (2\pi t - \nu ) \, , \, \xi \right\} , \quad t \ge 0. \end{aligned}$$

which allows incorporation of a trend and a given cyclic period with individual specific parameters, with $\xi$ a detectability threshold. This could be useful to plan trials involving nutrients with cumulative benefit, or supplements that are administered periodically (e.g. vitamin B12 shots or vitamin K antagonists).

In conclusion, understanding the population characteristics and trial features is key to accurately discerning the power of vitamin D trials.

## Supplementary Information

Supplementary Information 1.
