# Publisher Correction: Power determination in vitamin D randomised control trials and characterising factors affecting it through a novel simulation-based tool

**DOI:** 10.1038/s41598-021-92486-4

**Published:** 2021-06-22

**Authors:** Jason Wyse, Rebecca Mangan, Lina Zgaga

**Affiliations:** 1 Discipline of Statistics and Information Systems, School of Computer Science and Statistics, Trinity College Dublin, College Green, Dublin 2, Ireland; 2 Discipline of Public Health and Primary Care, Institute of Population Health, Trinity College Dublin, Tallaght Cross, Tallaght, Dublin 24 Ireland

Correction to: *Scientific Reports* 10.1038/s41598-021-90019-7, published online 24 May 2021

The original version of this Article contained an error in the Introduction section, where,

“The tools discussed in this paper are easily used through the undefined R package SimVitD^26^ available on the Comprehensive R Archive Network (CRAN).”

now reads:

“The tools discussed in this paper are easily used through the R package SimVitD^26^ available on the Comprehensive R Archive Network (CRAN).”

The original Article has been corrected.

