# Supplementary material for: Power determination in vitamin D randomised control trials and characterising factors affecting it through a novel simulation-based tool
Source: Sci Rep. 2021 May 24;11:10804. doi: 10.1038/s41598-021-90019-7 (PMC8144427; doi:10.1038/s41598-021-90019-7)
Supplement: Supplementary file 1 — Supplementary Information 1. [file 41598_2021_90019_MOESM1_ESM.pdf]

Supplementary material for "Power Determination in Vitamin D Randomised Control Trials and Characterising Factors Affecting it through a Novel Simulation-Based Tool" by Wyse, Mangan and Zgaga

S.1. Proof of Theorem 4.1

To make the argument more readable, we suppress the subscript i on a profile, but this is to be understood. The generative model for an individual status profile has $A \sim f(A)$, $H \sim f(H)$ where f denotes the corresponding density function, and $V^{\text{pl}}(t) = \max\{\mu + H + A\cos(2\pi t - \nu), 10\}$. Let $V^{\text{supp}}(t)$ denote the curve $V^{\text{pl}}(t)$ adjusted to reflect a particular supplementation scheme for individual i. For both the fixed and dynamic dose schedule $V^{\text{supp}}(t) \geq V^{\text{pl}}(t), t \geq 0$. Let Ω be the union of all (disjoint) intervals of $\mathbb{R}^+$ for which it is possible that $V^{\text{supp}}(t) = V^{\text{pl}}(t)$. Let $\Omega^c = \mathbb{R}^+ \backslash \Omega$, then $V^{\text{supp}}(t) > V^{\text{pl}}(t)$ for any $t \in \Omega^c$. This condition is satisfied by all supplementation schemes we discuss.

At time t, the probability of contracting infection from an exposure is given by the (general logistic) relative risk curve for the status level and multiplying by the baseline probability:

$$p(t) \quad = \quad p_0\, g\left(V(t)\right)$$

with $g(x)$ the generalised logistic curve describing relative risk scaling introduced in Section 2.3.2. Since $g(x)$ is strictly decreasing,

$$\begin{array}{lll} g\left(V^{\text{supp}}(t)\right) < g\left(V^{\text{pl}}(t)\right) & \Longrightarrow\; p^{\text{supp}}(t) < p^{\text{pl}}(t) & \text{for } t \in \Omega^c \\ g\left(V^{\text{supp}}(t)\right) = g\left(V^{\text{pl}}(t)\right) & \Longrightarrow\; p^{\text{supp}}(t) = p^{\text{pl}}(t) & \text{for } t \in \Omega \end{array} \tag{4}$$

Given a trial running over $(0, \tau]$, let the number of exposures be denoted $X(\tau)$ where $X(t), t \geq 0$ is a NHPP with intensity function $\lambda(t)$. At each exposure, whether an infection occurs or not is determined by outcomes of independent (conditional on A, H etc.) Bernoulli trials with success probability given by $p^{\text{pl}}(t)$, or $p^{\text{supp}}(t)$ for supplementation. The random variable counting total number of infections is

$$N^{\text{pl}}(\tau) \quad = \quad \sum_{l=1}^{X(\tau)} Z_{T_l}, \qquad Z_t \sim \text{Bernoulli}\left(p^{\text{pl}}(t)\right)$$

for the placebo case. The $T_1, \ldots, T_{X(\tau)}$ denote times of random exposures to infection as arrivals from the NHPP. Let $N^{\text{supp}}(\tau)$ be the analogous infection count for the supplementation scheme. Condition on the values of A, H and δ or ρ to begin. Denote these collectively as Ψ, and their density as $p(\Psi)$ (we assume they are marginally independent in our simulation model). The result applies to setups of practical interest, so in what follows we assume $p_0 > 0$, $\lim_{x \to -\infty} g(x) = 1$ and $\lambda(t)$ is non-zero on an interval of time of non-negligible length during the study.

Statement (i): Letting $\theta_s = \Pr\{\text{individual gets} \geq 1 \text{ infection in arm } s\}$, we have

$$\theta_s = 1 - \Pr\{\text{individual stays infection free in arm } s\}.$$

An individual staying infection free in arm s is equivalent to the event $N^s(\tau) = 0$. This happens only if no exposures result in infection, which happens with probability

$$\prod_{l=1}^{X(\tau)} \left(1 - p^s(T_l)\right).$$

Using a conditional probability decomposition

$$\begin{aligned} \Pr\{N^s(\tau) = 0 | \Psi\} &= \sum_{k=0}^{\infty} \Pr\{N^s(\tau) = 0 | X(\tau) = k, \Psi\} \Pr\{X(\tau) = k\} \\ &= \sum_{k=0}^{\infty} \left[\int_{\mathscr{A}} \Pr\{N^s(\tau) = 0 | T_1 = t_1, \ldots, T_k = t_k, \Psi\} f_{(0,\tau]}(t_1, \ldots, t_k)\, \mathrm{d}t_1 \ldots \mathrm{d}t_k\right] \Pr\{X(\tau) = k\} \\ &= \sum_{k=0}^{\infty} \left[\int_{\mathscr{A}} \prod_{l=1}^{k} (1 - p^s(t_l)) \times f_{(0,\tau]}(t_1, \ldots, t_k)\, \mathrm{d}t_1 \ldots \mathrm{d}t_k\right] \Pr\{X(\tau) = k\} \end{aligned}$$

where $f_{(0,\tau]}(t_1, \ldots, t_k)$ is the joint distribution of the first k arrival times $T_1, T_2, \ldots, T_k$ of an NHPP with intensity function $\lambda(t)$, conditional on $T_k \leq \tau$. Denote the region $t_1 < t_2 < \cdots < t_k$ by $\mathscr{A}$. Consider the product space $\Omega^k = \Omega \times \Omega \times \cdots \times \Omega$, such that

$(t_1,\ldots,t_k) \in \Omega^k$ if $t_j \in \Omega$ for $j = 1,\ldots,k$. Split the domain of integration $\mathscr{A}$ of the integral in the square brackets into that which intersects with Ω^k and its complement

$$\begin{aligned}
&\int_{\mathscr{A}} \prod_{\ell=1}^{k} (1-p^s(t_\ell)) \times f_{(0,\tau]}(t_1,\ldots,t_k)\,\mathrm{d}t_1\ldots\mathrm{d}t_k \\
&= \int_{\mathscr{A}\cap\Omega^k} \prod_{\ell=1}^{k} (1-p^s(t_\ell)) \times f_{(0,\tau]}(t_1,\ldots,t_k)\,\mathrm{d}t_1\ldots\mathrm{d}t_k \\
&\qquad + \int_{\mathscr{A}\cap(\Omega^k)^c} \prod_{\ell=1}^{k} (1-p^s(t_\ell)) \times f_{(0,\tau]}(t_1,\ldots,t_k)\,\mathrm{d}t_1\ldots\mathrm{d}t_k.
\end{aligned}$$

Considering the last integral in this expression. For a point $(t_1,\ldots,t_k) \in \mathscr{A} \cap (\Omega^k)^c$, there is at least one t_j for which $t_j \notin \Omega$ and we can write

$$\begin{aligned}
\prod_{l=1}^{k}(1-p^{\text{pl}}(t_l)) &= \prod_{\ell:t_\ell\in\Omega}(1-p^{\text{pl}}(t_l)) \prod_{\ell:t_\ell\notin\Omega}(1-p^{\text{pl}}(t_\ell)) \\
&< \prod_{\ell:t_\ell\in\Omega}(1-p^{\text{supp}}(t_\ell)) \prod_{\ell:t_\ell\notin\Omega}(1-p^{\text{supp}}(t_\ell)) = \prod_{\ell=1}^{k}(1-p^{\text{supp}}(t_\ell)).
\end{aligned}$$

Hence, $\Pr\{N^{\text{pl}}(\tau)=0|\Psi\} < \Pr\{N^{\text{supp}}(\tau)=0|\Psi\}$. Considering all possible values of Ψ:

$$\begin{aligned}
\Pr\{N^{\text{pl}}(\tau)=0\} &= \int \Pr\{N^{\text{pl}}(\tau)=0|\Psi\}\,p(\Psi)\,\mathrm{d}\Psi \\
&< \int \Pr\{N^{\text{supp}}(\tau)=0|\Psi\}\,p(\Psi)\,\mathrm{d}\Psi = \Pr\{N^{\text{supp}}(\tau)=0\}
\end{aligned}$$

implying $1-\Pr\{N^{\text{pl}}(\tau)=0\} > 1-\Pr\{N^{\text{supp}}(\tau)=0\}$ and thus $\theta_{\text{pl}} > \theta_{\text{supp}}$.

Statement (ii): In this case, to get $\mathrm{E}(N^{\text{pl}}(\tau)|\Psi)$ using a sequence of conditioning arguments gives,

$$\begin{aligned}
\mathrm{E}(N^{\text{pl}}(\tau)|\Psi) &= \sum_{k=0}^{\infty} \mathrm{E}(N^{\text{pl}}(\tau)\,|\,X(\tau)=k,\Psi)\,\Pr\{X(\tau)=k\} = \sum_{k=0}^{\infty} \mathrm{E}\left(\sum_{\ell=1}^{k} Z_{T_\ell}\middle|\Psi\right)\Pr\{X(\tau)=k\} \\
&= \sum_{k=0}^{\infty}\left[\int_{\mathscr{A}} \mathrm{E}\left(\sum_{\ell=1}^{k} Z_{T_\ell}\middle| T_1=t_1,\ldots,T_k=t_k,\Psi\right) f_{(0,\tau]}(t_1,t_2,\ldots,t_k)\,\mathrm{d}t_1\ldots\mathrm{d}t_k\right]\Pr\{X(\tau)=k\} \\
&= \sum_{k=0}^{\infty}\left[\int_{\mathscr{A}}\left(\sum_{\ell=1}^{k} p^{\text{pl}}(t_\ell)\right) f_{(0,\tau]}(t_1,t_2,\ldots,t_k)\,\mathrm{d}t_1\ldots\mathrm{d}t_k\right]\Pr\{X(\tau)=k\}
\end{aligned}$$

and using the same idea as above splitting the domain of integration

$$\sum_{\ell=1}^{k} p^{\text{pl}}(t_\ell) = \sum_{\ell:t_\ell\in\Omega} p^{\text{pl}}(t_\ell) + \sum_{\ell:t_\ell\notin\Omega} p^{\text{pl}}(t_\ell) > \sum_{\ell:t_\ell\in\Omega} p^{\text{supp}}(t_\ell) + \sum_{\ell:t_\ell\notin\Omega} p^{\text{supp}}(t_\ell) = \sum_{\ell=1}^{k} p^{\text{supp}}(t_\ell)$$

where the inequality follows from (4). Thus

$$\begin{aligned}
\mathrm{E}(N^{\text{pl}}(\tau)|\Psi) &= \sum_{k=0}^{\infty}\left[\int_{\mathscr{A}}\left(\sum_{\ell=1}^{k} p^{\text{pl}}(t_\ell)\right) f_{(0,\tau]}(t_1,t_2,\ldots,t_k)\,\mathrm{d}t_1\ldots\mathrm{d}t_k\right]\Pr\{X(\tau)=k\} \\
&> \sum_{k=0}^{\infty}\left[\int_{\mathscr{A}}\left(\sum_{\ell=1}^{k} p^{\text{supp}}(t_\ell)\right) f_{(0,\tau]}(t_1,t_2,\ldots,t_k)\,\mathrm{d}t_1\ldots\mathrm{d}t_k\right]\Pr\{X(\tau)=k\} = \mathrm{E}(N^{\text{supp}}(\tau)|\Psi).
\end{aligned}$$

Making explicit the conditioning on Ψ, it has been shown that $\mathrm{E}(N^{\text{pl}}(\tau)|\Psi) > \mathrm{E}(N^{\text{supp}}(\tau)|\Psi)$. Using the law of total expectation to average over all possible vitamin D status curves by marginalising over Ψ, the expected number of infections for placebo individuals in a study of length τ is

$$\int \mathrm{E}(N^{\text{pl}}(\tau)|\Psi)\;p(\Psi)\,\mathrm{d}\Psi > \int \mathrm{E}(N^{\text{supp}}(\tau)|\Psi)\;p(\Psi)\,\mathrm{d}\Psi$$

and $\mu_{\text{pl}} > \mu_{\text{supp}}$.

S.2. Truncated distribution

The distribution assumed for the individual level increases in the fixed increase scheme is an exponential distribution reflected about the administered increase δ truncated at 0 and δ. Its density and distribution function are

$$f(t) = \frac{\gamma \mathrm{e}^{-\gamma(\delta - t)}}{1 - \mathrm{e}^{-\gamma\delta}} \mathbb{I}(0 < t < \delta) \qquad F(t) = \frac{\mathrm{e}^{-\gamma(\delta - t)} - \mathrm{e}^{-\gamma\delta}}{1 - \mathrm{e}^{-\gamma\delta}}$$

where the parameter γ can be used to control the spread close to δ. The first moment is given by $\frac{\delta}{1-\mathrm{e}^{-\gamma\delta}} - \frac{1}{\gamma}$. Random generation can be carried out straightforwardly by inverting the cumulative distribution function. If $U \sim \mathrm{Uniform}(0,1)$ then

$$\delta + \frac{1}{\gamma} \log \left[\mathrm{e}^{-\gamma\delta} + (1 - \mathrm{e}^{-\gamma\delta}) U \right]$$

is a sample from $F(\cdot)$. Choosing a large value of γ concentrates all derived increases around δ. This is the choice made in Sections 2.5.1 and 2.5.2.

S.3. Glossary of parameters

Parameter	Explanation	Section 2.5
(μ_A, σ_A)	Mean and standard deviation of simulated individual amplitudes	$(15, 5)$
(μ, σ_H)	Height and standard deviation of the height perturbation	(Many, 5)
(δ, γ)	Maximum administered dose and uptake concentration around that dose	(Many, ∞)
(μ_ρ, σ_ρ)	Mean and standard deviation of the individual level in concentration controlled scheme	Not used here
$(\mu_\omega, \sigma_\omega)$	Mean and standard deviation of proportion of equivalent dose utilised all year around	$(0.8, 0.1)$
$(\tau_{\text{start}}, \tau_{\text{end}})$	Duration of study where start is counted from beginning of March	Many used
$\lambda(t)$	Rate function of IHPP for exposures	Figure 2
p_0	Baseline risk of adverse event given exposure has occurred when sufficient	0.03
$g(x)$	Generalised logistic function describing scaling of baseline risk for different sufficient levels	Most change between (10,70) nmol/L
(ℓ, u)	Risk scale extremes	(1, 2 or 4)
(n, r)	The number of participants in the placebo group and the corresponding fold factor for the number in the tratment group	(Many, 1)
N	Number of independent simulations of the trial	500
α	Significance level for the test used	0.05

Code provided with the supplementary material reproduces the experiments reported in Section 2.5. The table above gives a break down of the choices made (where appropriate). We argue that Vitamin D investigators should think about the mechanistic parameters above when planning a trial. It provides a route to envisaging potential sources of variability in trials which might impact power.

S.4. Further experimental results for one year trial

This section gives more verbose reporting of the simulation experiments. Designs achieving $\geq 80\%$ power are shown in bold font. Here it can be observed that when the supplementation is substantial enough, effects might be detectable at smaller n in the vitamin D deficient group (15 nmol/L baseline). For those that are highly sufficient, supplementation makes no detectable difference.

n (per arm)	15 nmol/L	35 nmol/L	50 nmol/L	60 nmol/L	75 nmol/L
100	8.0	15.5	15.1	11.2	6.4
200	11.6	21.0	20.3	14.1	7.2
300	11.0	27.3	26.1	16.5	7.4
400	13.9	33.6	32.2	21.1	9.0
500	14.6	38.8	38.3	23.8	10.2
600	16.2	43.3	42.2	26.1	9.8
700	17.5	49.3	47.1	28.8	10.1
800	18.5	54.1	52.6	34.7	12.8
900	20.0	59.0	57.1	35.6	11.9
1000	21.9	63.1	59.3	36.7	12.5
1100	25.1	68.0	65.2	42.2	13.8
1200	23.9	69.2	66.9	44.0	13.6
1300	26.6	75.0	72.3	46.8	14.9
1400	27.2	75.0	72.9	48.2	14.7
1500	28.2	78.2	75.6	50.0	14.8

Table S1. Power (in percentage) for 10 nmol/L increase equivalent dose at $u = 2$ for a range of sample sizes and baseline population 25OHD concentrations.

n (per arm)	15 nmol/L	35 nmol/L	50 nmol/L	60 nmol/L	75 nmol/L
100	67.0	71.8	40.2	20.6	8.160
200	**89.2**	**92.3**	64.4	31.5	10.2
300	**97.0**	**98.4**	77.9	39.7	10.7
400	**99.4**	**99.6**	**86.9**	48.3	13.3
500	**99.9**	**100.0**	**93.1**	57.6	15.0
600	**100.0**	**100.0**	**97.1**	62.8	14.7
700	**100.0**	**100.0**	**98.3**	69.8	16.4
800	**100.0**	**100.0**	**99.3**	74.2	16.7
900	**100.0**	**100.0**	**99.3**	79.9	19.0
1000	**100.0**	**100.0**	**99.6**	**83.9**	20.4
1100	**100.0**	**100.0**	**99.8**	**85.6**	22.7
1200	**100.0**	**100.0**	**99.9**	**88.2**	23.5
1300	**100.0**	**100.0**	**100.0**	**90.3**	23.2
1400	**100.0**	**100.0**	**100.0**	**92.4**	24.5
1500	**100.0**	**100.0**	**100.0**	**94.2**	25.7

Table S2. Power (in percentage) for 40 nmol/L increase equivalent dose at $u = 2$ for a range of sample sizes and baseline population 25OHD concentrations.

n (per arm)	15 nmol/L	35 nmol/L	50 nmol/L	60 nmol/L	75 nmol/L
100	14.4	41.5	44.5	30.8	11.8
200	21.6	64.1	68.6	47.3	16.8
300	27.1	79.7	**82.8**	62.2	21.0
400	31.1	**89.6**	**91.7**	73.0	22.4
500	40.0	**93.5**	**96.1**	**81.3**	25.9
600	44.3	**96.8**	**97.6**	**85.7**	30.1
700	51.8	**98.8**	**99.2**	**91.7**	34.6
800	53.1	**99.1**	**99.7**	**94.8**	36.5
900	58.6	**99.4**	**99.8**	**96.4**	40.5
1000	62.5	**99.8**	**99.9**	**97.9**	41.6
1100	66.1	**99.9**	**100.0**	**98.6**	44.1
1200	68.6	**100.0**	**100.0**	**99.1**	47.5
1300	71.2	**100.0**	**100.0**	**99.7**	51.1
1400	73.5	**100.0**	**100.0**	**99.7**	52.8
1500	76.6	**100.0**	**100.0**	**99.9**	54.5

Table S3. Power (in percentage) for 10 nmol/L increase equivalent dose at $u = 4$.

n (per arm)	15 nmol/L	35 nmol/L	50 nmol/L	60 nmol/L	75 nmol/L
100	49.2	**91.6**	**84.2**	56.2	16.00
200	75.3	**99.4**	**98.1**	**81.4**	22.9
300	**89.5**	**100.0**	**99.9**	**94.0**	30.5
400	**95.0**	**100.0**	**100.0**	**97.3**	35.6
500	**97.8**	**100.0**	**100.0**	**99.2**	43.5
600	**99.5**	**100.0**	**100.0**	**99.8**	48.9
700	**99.5**	**100.0**	**100.0**	**99.9**	54.2
800	**100.0**	**100.0**	**100.0**	**100.0**	58.8
900	**99.9**	**100.0**	**100.0**	**100.0**	64.1
1000	**100.0**	**100.0**	**100.0**	**100.0**	67.4
1100	**100.0**	**100.0**	**100.0**	**100.0**	71.2
1200	**100.0**	**100.0**	**100.0**	**100.0**	74.6
1300	**100.0**	**100.0**	**100.0**	**100.0**	76.3
1400	**100.0**	**100.0**	**100.0**	**100.0**	79.1
1500	**100.0**	**100.0**	**100.0**	**100.0**	81.4

Table S4. Power (in percentage) for 20 nmol/L increase equivalent dose at $u = 4$.

n (per arm)	15 nmol/L	35 nmol/L	50 nmol/L	60 nmol/L	75 nmol/L
100	**100.0**	**100.0**	**98.0**	74.2	18.76
200	**100.0**	**100.0**	**100.0**	**94.4**	26.6
300	**100.0**	**100.0**	**100.0**	**98.7**	37.8
400	**100.0**	**100.0**	**100.0**	**99.7**	47.6
500	**100.0**	**100.0**	**100.0**	**100.0**	53.2
600	**100.0**	**100.0**	**100.0**	**100.0**	58.9
700	**100.0**	**100.0**	**100.0**	**100.0**	65.8
800	**100.0**	**100.0**	**100.0**	**100.0**	71.3
900	**100.0**	**100.0**	**100.0**	**100.0**	74.1
1000	**100.0**	**100.0**	**100.0**	**100.0**	78.6
1100	**100.0**	**100.0**	**100.0**	**100.0**	**80.7**
1200	**100.0**	**100.0**	**100.0**	**100.0**	**86.2**
1300	**100.0**	**100.0**	**100.0**	**100.0**	**87.6**
1400	**100.0**	**100.0**	**100.0**	**100.0**	**89.8**
1500	**100.0**	**100.0**	**100.0**	**100.0**	**91.4**

Table S5. Power in percentage for 40 nmol/L equivalent dose at $u = 4$.

S.5. Power in published studies

This section details an illustrative retrospective calculation of power in some studies which have examined the benefits of supplementation in prevention of respiratory tract infections in various populations. These studies were identified from the broad and thorough IPD (individual patient data) meta-analysis by Martineau et al. (2017)[42]. We took a varied selection of studies from the IPD where vitamin D supplementation was administered daily.

For calculation using our proposed generative framework, some settings are kept the same across all studies. The Poisson exposures process is identical to that shown in Figure 2. We consider $u = 2,4$ with $l = 1$, to reflect low/high dose response situations. For all experiments we assume that $\mu_A = 15, \sigma_A = 5$ which gives a 95% interval of $(6.9, 26.3)$nmol/L fluctuations of an individual's 25OHD around their individual mean $(\mu + H_i)$ level. A value of $\gamma = \infty$ is applied in all cases, so that the entire equivalent of every dose is absorbed by every participant. We assume that supplementation in winter gives slightly more benefit $\mu_\omega = 0.8, \sigma_\omega = 0.1$ as described in Section 2.2.1. A value of $p_0 = 0.03$ is used for all studies. There was ten replications of $N = 1000$ trials simulated in each instance. A Type I error rate of $\alpha = 0.05$ was used for all studies.

In determining δ (the expected increase in 25OHD from supplementation) we make what we believe to be a reasonable but conservative conversion from μg daily to nmol/L, based on available literature. This conversion is an engaging calibration problem in and of itself which encourages debate. Where participants are enrolled in a staggered fashion, we take chose a single start date that would maximise the power as much as possible.

Before describing the studies considered in further detail, we'd like the reader to note that inclusion of these specific experiments is for a demonstrative purpose in this instance. A full post–hoc analysis would require a much more careful and rigorous treatment to establish equivalence in parameter choices. Our aim here is to highlight the many different scenarios and constraints that investigators work under, and how situational and temporal factors may impact power in a broad sense. We are

aware of the blood, sweat and tears involved in conducting any trial, and aware too of how a modelling tool like that which we propose cannot reflect all facets of such an involved endeavour. However, we believe our tool will give a good reflection of patterns of variability in broad brush-stroke terms for what is, effectively, a complex system. Patterns which we believe to be nigh on impossible to capture through methods requiring an investigator to specify just two point values and an expected effect size, as would be done when using out-of-the-box sample size determination methods. In a prospective planning scenario, we would envisage our tool working symbiotically with expert opinion during the trial set up, so that rather than power calculations being a task exclusively for a supporting statistician, a conversation invites investigators to probe sources of variability in the trial context. A more nuanced set of parameter values than those we assume here would result from such a process.

Li-Ng (2009)[44]: This was a 3 month trial. Recruitment was staggered from December 2006 to March 2007 and the study ran until June 2007. The recruitment was through newspaper adverts, flyers at medical offices etc. with a wide age range in the inclusion criteria. Participants either received 50μg/day (2000 IU/day) supplement or placebo with treatment randomly assigned. Baseline 25OHD level was 63.7 ± 25.5 nmol/L. In their discussion of power the authors state that initially the study was powered to detect a 25% difference in incidence of URIs in the placebo and treatment arms, but taking into account attrition they could conclude 80% power to detect a 23% difference in URI incidence between arms. This power calculation appears to be based on a two independent samples proportions test.

Laaksi (2010)[45]: This was a 6 month trial. The trial began in October 2005 and ran until March 2006. Participants were young men undergoing compulsory military service. Participants received 10μg/day (400 IU/day) supplement or placebo with treatment randomly assigned. Baseline 25OHD level was 75.9 ± 18.7 nmol/L. Power calculations were not carried out, the investigators recruited all volunteers possible.

Bergman (2012):[46]: This was a one year trial with participants recruited from an immunodeficiency unit between March and June (2010). The primary endpoint in this study was an infectious score which is different to the other trials. Baseline 25OHD was 49.3 ± 23.2 nmol/L. A sample size calculation was carried out using the expected number of days with symptoms: these would be reduced from 42 to 28 days in the supplementation group. The authors used a target of 90% power at a Type I error rate of 0.02. The distributional assumption used to calculate the power was stated as the Student's t test.

Urashima (2014):[47] This trial took place over two months from October to December 2009 in Japan. Participants were high school students. In this case, the investigators did not want to deter volunteers from participating by carrying out an invasive blood test, so that 25OHD was not measured at recruitment. We use conservative values for the study regarding the 25OHD generative characteristics. The sample size was calculated by assuming that supplementation would be sufficient in reducing incidence by 60%, where the overall prevalence was assumed to be 25% to begin with. This sample size calculation was carried out with a two sample proportions calculation, two tailed, with a Type I error rate of 0.05. The authors conclude their study has 85% power (we use the 1:1 ratio, diving 254 by two).

Table S6 outlines the results of the comparative study. The reported power in the original publication, when available, is usually higher except in one case. The count test (test (2) in our paper) will be more highly powered than the proportions (test (3) in our paper). The reason for this is that counts (ie. number of infections) provide a more granular record of the trial outcomes (compared to any infections: yes/no). The risk scaling u has a huge impact on power when increased from 2 to 4. This is not surprising, in that if the benefit of vitamin D does not manifest strongly in observable measurements, then a large sample size will be required to detect a difference between groups. Note that the 25OHD for Urashima et al. (2014)[47] were chosen by us. They are slightly lower than the other studies, and paired with the larger sample size than the other studies, this leads to the higher power values observed.

Parameters	Li-Ng (2009)	Laaksi (2010)	Bergman (2012)	Urashima (2014)
$(\tau_{\text{start}}, \tau_{\text{end}})^{\dagger}$	$(0, \frac{3}{12})$	$(\frac{7}{12}, 1)$	$(0, 1)$	$(\frac{7}{12}, \frac{9}{12})$
μ	63.7	75.9	49.3	45.0
σ_H	25.5	18.7	23.2	20.0
Dose	50μg/day	10μg/day	100μg/day	50μg/day
n determined	85	84	60	127
Power reported	80%	ND	90%	85%
α reported	0.05	ND	0.02	0.05
`SimVitD` δ dose nmol/L	40	20	60	40
Count test (2): power $u = 2$	8.7%	5.3%	14.7%	25.6%
$u = 4$	34.5%	15.2%	78.4%	90.9%
effect $u = 2$	0.03	0.04	0.16	0.10
$u = 4$	0.10	0.10	0.48	0.29
Prop. test (3): power $u = 2$	8.1%	4.0%	8.0%	19.5%
$u = 4$	28.2%	8.8%	49.4%	78.3%
effect $u = 2$	0.03	0.02	0.09	0.06
$u = 4$	0.08	0.06	0.22	0.17

Table S6. Table showing settings and results of retrospective power calculation for studies identified in the IPD of Martineau et al. (2017)[42]. The n determined gives a number per trial arm. In the case of Laaksi et al. we used the larger of the two group sizes. The power and effect size through simulation for tests (2) and (3) in main manuscript are given. Note†: within the `SimVitD` package[26], March corresponds to time zero.
